# SERIAL MEDIATION BY DIGITAL ASSISTANCE, INTERNET SELF-EFFICACY, DIGITAL CITIZENSHIP, AND UNMET NEEDS

**DOI:** 10.1093/geroni/igac059.2191

**Published:** 2022-12-20

**Authors:** Susanna Joo, Bomi Choi, Hayoung Park, Changmin Lee, Yoon-Myung Kim, Hyoun K Kim

**Affiliations:** Yonsei University, Seoul, Seoul-t'ukpyolsi, Republic of Korea; Yonsei University, Seoul, Seoul-t'ukpyolsi, Republic of Korea; Yonsei University, Seoul, Seoul-t'ukpyolsi, Republic of Korea; Yonsei University, Seoul, Seoul-t'ukpyolsi, Republic of Korea; Yonsei University, Seoul, Seoul-t'ukpyolsi, Republic of Korea; Yonsei University, Seoul, Seoul-t'ukpyolsi, Republic of Korea

## Abstract

Although many social services are getting digitalized with the rapidly growing digital technology, there is little information available on the specific process of meeting personal needs in digital aging contexts. The present study examined the serial mediation model from digital assistant to unmet needs via internet self-efficacy and digital citizenship. An online survey was used to collect data in December 2021, and the sample included 223 older Korean adults aged 65 and above (M=68.79, SD=4.18, range=65-84) who owned at least one digital device. Unmet needs, the dependent variable, was the number of problems (e.g., housing, physical functioning) that participants responded to as ‘unmet.’ The independent variable was digital assistance, meaning getting help from friends or others when having problems with the digital device. The first mediator was internet self-efficacy, and the second mediator was five subtypes of digital citizenship (internet political activism, technical skills, local/global awareness, critical perspective, and networking agency). Covariates were gender, age, education, and income. SPSS Process Macro was utilized for serial mediation analysis. The result showed that only a serial mediation path from digital assistance to unmet needs via internet self-efficacy and internet political activism was significant. Additionally, a simple mediation path from digital assistance to unmet needs via internet political activism was significant. Technical skills, local/global awareness, critical perspective, and networking agency did not reveal significant mediational paths. Findings imply providing digital assistance system may be useful to reduce unmet needs among older adults by enhancing self-efficacy and political participation in the current digitalized world.

